# Identification and Functional Characterization of a Low-Density Lipoprotein Receptor Gene Pathogenic Variant in Familial Hypercholesterolemia

**DOI:** 10.3389/fgene.2021.650077

**Published:** 2021-08-23

**Authors:** Hong-Yan Shu, Wei Zhang, Cong-Cong Zheng, Man-Yun Gao, Yong-Cun Li, Yan-Gang Wang

**Affiliations:** ^1^Department of Endocrinology and Metabolic Diseases, Zibo Municipal Hospital, Zibo, China; ^2^Department of Endocrinology and Metabolic Diseases, The Affiliated Hospital of Qingdao University, Qingdao, China

**Keywords:** low-density lipoprotein receptor gene, genomic variant, familial hypercholesterolemia, functional analyses, Golgi apparatus

## Abstract

We report a single-point variant of low-density lipoprotein receptor (*LDLR*) in a Chinese proband with a clinical diagnosis of familial hypercholesterolemia (FH) with a comprehensive functional analysis. Target exome capture-based next-generation sequencing was used for sequencing and identification of genomic variants in the *LDLR* gene. The expression, cellular location, and function of the mutant LDLR were analyzed. Sequencing of *LDLR* in FH patients indicated a point variant of single-base substitution (G < A) at a position of 2389 in the 16th exon, which led to a loss of the 16th exon in the *LDLR* messenger RNA. This genomic variant was found to cause exon 16 deletion in the mutant LDLR protein. Subsequent functional analyses showed that the mutant LDLR was retained in the Golgi apparatus and rarely expressed in the cellular membranes of HepG2 cells. Accordingly, the intake ability of HepG2 cells with the mutant LDLR was significantly reduced (*P* < 0.05). In conclusion, our results suggest that a mutant with a single-base substitution (c. 2389G > A) in the 16th exon of the *LDLR* gene was associated with miscleavage of messenger RNA and the retention of mutant LDLR in the Golgi apparatus, which revealed a pathogenic variant in LDLR underlying the pathogenesis of FH.

## Introduction

Family hypercholesterolemia (FH) is an inherited disease that mainly affects the metabolism of cholesterol (Watts et al., 2020). Clinically, FH is characterized by significantly raised serum levels of total cholesterol (TC) and low-density lipoprotein cholesterol (LDL-C), the presence of tendinous xanthomata, and high incidence of premature vascular disease, particularly coronary artery disease (Garg et al., 2019; Hamasaki and Kotani, 2020). The serum level of TC can be as high as 7–15 mmol/L in patients with heterozygous FH, whereas the level of TC can reach 20–25 mmol/L in patients with homozygous FH (Santos et al., 2016). The prevalence of heterozygous FH has been reported to be 1/500 (0.2%), whereas the prevalence of homozygous FH has been reported to be 1/1,000,000 (Reiner, 2015). However, this prevalence is thought to be underestimated. In a study of the general Danish population in 2012, the prevalence of FH was reported to be 1/200 (0.5%) (Benn et al., 2012), which is similar to the results of the National Health and Nutrition Examination Surveys conducted in the United States between 1999 and 2012 (de Ferranti et al., 2016). A study based on the Chinese adult population showed a prevalence of FH of 0.47% in 2014, which is similar to values in western populations (Shi et al., 2014; Zhou and Zhao, 2016). More importantly, comorbidities of FH have been associated with poor prognosis, particularly premature cardiovascular death. A previously published study in Norway showed that cardiovascular mortality in patients with FH was significantly higher as compared with that among patients without FH (standardized mortality ratio: 2–3, despite the use of statins; Mundal et al., 2014). Treatment strategies for FH include statins, which lower the LDL-C level in the short term, but the long-term efficacy of statin treatment in patients with FH, particularly related to cardiovascular outcomes, remains to be determined (Vuorio et al., 2017). Therefore, continuous efforts are needed to improve the diagnosis and treatment of FH.

Pathophysiologically, FH is caused by genomic variants of key genes that are involved in the metabolism of cholesterol, including the low-density lipoprotein receptor (*LDLR*), the proprotein convertase subtilisin/kexin type 9 (*PCSK9*), and the apolipoprotein B (*APOB*) genes (Henderson et al., 2016; Sharifi et al., 2017; Vrablik et al., 2020). Of note, genomic variants in *LDLR* are considered the most important pathogenic mechanism in FH. LDLR is a transmembrane protein that functions in the uptake and removal of LDL-C from circulation. Many pathogenic variants have been reported to be involved in the pathogenesis of FH. A recent meta-analysis including Chinese patients with FH reported 131 genomic variants in *LDLR*, mostly allocated in exon 4 as missense variants, which may participate in the pathogenesis of FH (Jiang et al., 2015). Overall, more than 2,000 genomic variants have been reported in *LDLR* (Chemello et al., 2021), and these are classified into five categories (Soutar and Naoumova, 2007; Usifo et al., 2012; Hendricks-Sturrup et al., 2020). Class 1 variants represent *LDLR* genomic variants that lead to the prevented synthesis of immunodetectable LDLR protein. Class 2 variants indicate *LDLR* variants that cause complete (2a) or partial (2b) retainment of LDLR protein in the endoplasmic reticulum (ER). Class 3 variants cause the membrane to incorporate LDLR, but they are unable to bind LDL. Class 4 variants of *LDLR* consist of mutant *LDLR* that are unable to concentrate in clathrin-coated pits. Class 5 variants of *LDLR* present mutant LDLR that are unable to recycle LDL into the endosome. However, it has been noted that not all of the genomic variants in *LDLR* are associated with FH, which highlights the importance of functional characterization of *LDLR* genomic variants (Henderson et al., 2016; Lee, 2017; Sharifi et al., 2017).

In this study, we reported a genomic variant of *LDLR* that contributes to the pathogenesis of FH. Subsequent functional analysis showed a single-point genomic variant led to a miscleavage of messenger RNA (mRNA) and the retaining of mutant LDLR in the Golgi apparatus.

## Materials and Methods

### Ethical Approval

The protocol for this study was reviewed and approved by the Ethics Committee of The Affiliated Hospital of Qingdao University before its performance. All procedures performed in studies involving human participants were in accordance with the ethical standards of the institutional and national research committee and with the 1964 Declaration of Helsinki and its later amendments or comparable ethical standards. We obtained written informed consent from the patient for publication of identifying information/images in an online, open-access publication.

### Patient and Diagnostic Criteria of Family Hypercholesterolemia

The patient was diagnosed with FH at The Affiliated Hospital of Qingdao University. We used the Dutch Lipid Clinic Network diagnostic criteria for the diagnosis of FH (Catapano et al., 2016). Briefly, the Dutch Lipid Clinic Network criteria evaluate the possibility for the diagnosis of FH based on scores for the following four aspects: (1) family history of premature vascular disease, LDL-C level above the 95th percentile, presentation of tendinous xanthoma and/or arcus cornealis in a first-degree relative, or LDL-C level above the 95th percentile in a child < 18 years of age; (2) clinical presentation of the patient with premature vascular disease or LDL-C level above the 95th percentile; (3) physical examination of the patient showing tendinous xanthomata and/or arcus cornealis; (4) degree of LDL-C elevation of at least 4.0 mmol/L; and (5) DNA analyses showing functional genomic variants in the *LDLR*, *APOB*, or *PCSK9* gene. The diagnosis of FH is considered definite if the total score is over 8.

### Measurement of Blood Lipids

We obtained peripheral venous blood samples from the proband and her first-degree relatives in a fasting condition for the measurement of TC, LDL-C, triglycerides, and high-density lipoprotein cholesterol. Measurements of the lipid parameters mentioned earlier were performed with a chemiluminescence method with an automated biochemistry analyzer (Beckman AU 4500, United States) in The Affiliated Hospital of Qingdao University.

### Target Exome Capture-Based Next-Generation Sequencing of *LDLR* Gene

Genomic DNA of the proband, as well as the family members, was extracted from the peripheral venous blood sample with the phenol–chloroform centrifugation method as previously described (Schiebelhut et al., 2017). After treatment with ethylenediaminetetraacetic acid, genomic DNA samples of the proband were sent for customized target exome capture-based next-generation sequencing of the *LDLR*, *PCSK9*, and *APOB* genes at the Beijing Genomic Institution. The samples were sequenced simultaneously and analyzed for genomic variants of *LDLR* on the Illumina Platform, and a single-base variant within exon 16 was detected. Then, polymerase chain reaction (PCR) was performed targeting the mutated exome (exon 16) for the proband, her father, and paternal aunt and uncle (Table 1). Briefly, the primers were designed with Prime 5 as 5′-CCTTCCTTTAGACCTGGGCCT-3′ and 5′-CATAGCGGGAGGCTGTGACC-3′. We used 100 ng of genomic DNA as the template, which was mixed to obtain a total 50-μl reaction system using the HotStarTaq Plus DNA Polymerase Kit (Qiagen, Germany). The reactive conditions were as follows: Taq polymerase activation at 94±C for 5 min, 35 cycles of denaturing at 94±C for 30 s, annealing at 61–64±C for 30 s, extension at 72±C for 45 s, and final extension at 72±C for 5 min. We used agarose gel to separate and confirm the amplified products by electrophoresis. The PCR products were further purified using the DNA fragment purification kit (TaKaRa, Japan). Sequencing of the products was performed at the Beijing Genomic Institution with the Sanger sequencing method.

**TABLE 1:** Results of lipids analyses at admission for the proband and her relatives.

**Patient**	**Sex**	**Age, years**	**TG**	**TC**	**HDL-C**	**LDL-C**	**Multiple xanthomas**	**Arcus cornea**	**Statin use before diagnosis**	**Genomic variants**
Father	Male	50	1.96	9.3	0.71	6.45	Yes	No	Unknown	c. 2389G > A
Mother	Female	47	0.59	4.24	1.17	2.17	No	No	Unknown	Normal
Brother	Male	17	0.45	3.89	1.04	1.95	No	No	Unknown	Normal
Paternal uncle	Male	52	1.37	6.71	0.88	4.06	Yes	No	Unknown	c. 2389G > A
Paternal aunt	Female	43	2.41	11.93	1.17	7.79	Yes	No	Unknown	c. 2389G > A
Proband	Female	19	0.77	9.34	1.31	7.88	Yes	No	Occasionally	c. 2389G > A

*TG, triglyceride; TC, total cholesterol; HDL-C, high-density cholesterol; LDL-C, low-density cholesterol.*

### Reverse Transcription Polymerase Chain Reaction

Total RNA from the peripheral venous blood samples obtained from the proband and an age- and sex-matched health control was extracted using the Trizol method as previously described (Kim et al., 2017). Then, reverse transcription PCR was performed to evaluate the transcription of exon 16 with the primers designed by Primer 5 software, forward: 5′-TGAA CTGGTGTGAGAGGACC-3′, and reverse 5′-CTGGTTGTGG CAAATGTGGA-3′. After electrophoresis with agarose gel, the targeted bands were confirmed and separated, and the purified PCR products were sequenced with the Sanger sequencing method at the Beijing Genomic Institution.

### Construction of Mutant and Wild-Type LDLR Lentiviral Vectors

The construction of lentiviral vectors containing the coding regions of the mutant *LDLR* mentioned earlier at exon 16 and the wild-type (WT) *LDLR* genes, both with a merged FLAG protein on the C-terminus, was performed by the GeneChem Biotech Company (Shanghai, China). A GV416 plasmid with *Bam*HI enzymatic sites was used when preparing the vector, and the vectors were labeled with a green fluorescent protein. 293T cells were used to measure the supernatant virus titer.

### Culture of HepG2 Cells and Lentiviral Vector Transfection

HepG2 cells were purchased from YRgene Biotech Company (Shanghai, China) and cultured with Roswell Park Memorial Institute 1640 medium (Gibco, United States) supplemented with 10% fetal bovine serum, 100 units/ml penicillin, and 100 μg/ml streptomycin at 37±C in a humidified atmosphere at 5% carbon dioxide. For vector transfection, HepG2 cells were plated in six-well culture plates and infected with the lentiviruses carrying the WT *LDLR* or mutant *LDLR* with Polybrene Reagent (Invitrogen Life Technologies, Spain) according to the manufacturer’s instructions. Infected cells were maintained in culture for 72 h to achieve the maximal *LDLR* expression, and then the cells were cultured in a medium with Puromycin (1 μg/ml) to exclude the non-transfected cells.

### Analysis of Surface Biotinylated Proteins and Western Blotting

The membrane proteins of HepG2 cells were biotinylated and extracted as previously described (Whitaker et al., 2007). Briefly, HepG2 cells were washed with ice-cold phosphate-buffered saline (PBS) and incubated with buffer A [1 mg/ml Sulfo-NHS-(LC)-biotin in PBS, containing 1-mM MgCl_2_ and 1.3-mM CaCl_2_, pH 8.0] for 45 min at 4±C. Then, the cells were washed with cold PBS three times, scraped down, and centrifuged. After the cells were cultured in 300-μl buffer B (1% Triton X-100, 4-mM egtazic acid, 10-mM Tris, pH 8.0), they were lysed with ultrasonication, and the lysates were clarified by centrifugation for 15 min at 20,000 × *g* at 4±C. Then, 500-μg total protein was taken for each sample, and buffer B was added to a total volume of 280 μl before the protein sample was quantified. After the addition of 30 μl of a 50% slurry of High Capacity NeutrAvidin Agarose Resin (Pierce, United States) followed by mixing for 1 h at room temperature, the biotinylated proteins were precipitated from 280 μl of lysate. Then, the biotinylated proteins binding to the agarose were washed three times with ice-cold PBS and eluted from NeutrAvidin agarose after the addition of 20 μl of 2 × sodium dodecyl sulfate (SDS)-polyacrylamide gel electrophoresis sample buffer. Then, 20 μl of 50-mM dithiothreitol in SDS sample buffer was added in each sample and centrifuged at room temperature, and Bromophenol Blue was added and mixed before centrifugation for 1 min at 10,000 × *g* at 4±C. Then, the samples were used for Western blotting, with 500 μg of total protein loaded for each sample on 10% SDS-polyacrylamide gel electrophoresis gels, transferred to polyvinylidene difluoride membranes, and immunoblotted for FLAG proteins. All experiments for each sample were repeated at least three times.

### Confocal Laser Scanning Microscopy and 1,19-Dioctadecyl-3,3,3939-Tetramethylindocarbocyanine Perchlorate-Low-Density Lipoprotein Intake

Confocal laser scanning microscopy (CLSM) was used to evaluate the colocalization of LDLR-FLAG fusion proteins in the HepG2 cells. Briefly, the transfected cells with WT or mutant *LDLR* were seeded on coverslips, fixed with 4% paraformaldehyde for 15 min, and washed three times with ice-cold PBS. Then, cells were permeabilized with 1% Triton X-100 for 30 min at room temperature. The samples were washed and blocked with PBS-5% bovine serum albumin for 1 h. After washing three times, the samples were incubated with tetramethylrhodamine-conjugated concanavalin A (1:100, Molecular Probes, United States) at room temperature for 1 h. Then, the cells were washed three times with PBS and fixed with 4% paraformaldehyde for 15 min at room temperature for observation of the intracellular fluorescent dye by CLSM (Olympus IX 81, Tokyo, Japan). The intracellular fluorescent dye was observed with sequential excitation and capture image acquisition with a digital camera (Axiocam NRc5; Zeiss, Jena, Germany). We used the Fluoview v50 software (Olympus, Miami, FL, United States) to obtain the images, and quantitative analyses of the fluorescence intensities were performed with ImageJ software (NIH, Bethesda, MD, United States). Finally, the intracellular fluorescent dye was observed by CLSM.

To evaluate the LDL uptake ability of the cells, the HepG2 cells transfected with the mutant and WT *LDLR* were incubated with 20 mg/ml fluorescent 1,19-dioctadecyl-3,3,3939-tetramethylindocarbocyanine perchlorate (Dil)-conjugated LDL (Molecular Probes) in serum-free media and kept at 37±C for 4 h. The Golgi apparatus was labeled with anti-syntaxin 6. Then, the medium was removed, and the cells were washed with PBS three times. After fixation with 4% paraformaldehyde for 15 min at room temperature, the intracellular fluorescent dye, which is reflective of the uptake ability of LDL, was observed by CLSM.

### Statistical Analyses

We used the GraphPad Prism 6 (GraphPad Software, San Diego, CA) and SPSS 16.0 software programs (SPSS, Inc., Chicago, IL, United States) for statistical analyses. Data are presented as the mean ± standard error of the mean. The Student’s *t*-test was used for comparisons between two groups. A *P* < 0.05 was considered to be statistically significant.

## Results

### Clinical Characteristics of the Proband and Her First-Degree Relatives

**FIGURE 1:**
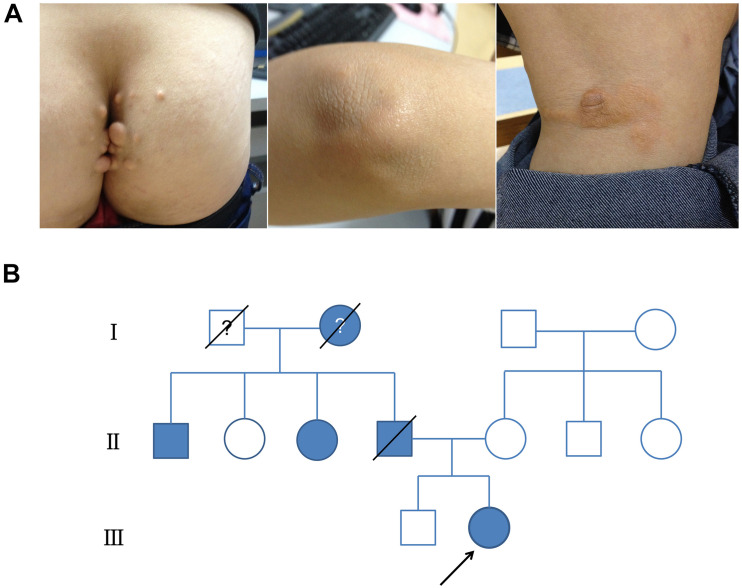
Clinical presentation and family tree of proband. **(A)** Physical examination revealed multiple xanthomas on buttocks, tendon, and popliteal space on proband. **(B)** Family tree showing confirmed hypercholesterolemia in family of proband (colored in gray).

The proband was a 19-year-old female who presented at our hospital with high levels of TC and LDL-C and multiple xanthomas on the buttocks that had been present she was 9 years old. Physical examination showed multiple xanthomas on the buttocks, tendon, and popliteal space of the proband (Figure 1A). As for the proband’s family history, her father had died at 48 years of age due to myocardial infarction, and he also had hypercholesterolemia and multiple tendon xanthomas. Moreover, one paternal aunt and one paternal uncle of the proband also had hypercholesterolemia and multiple tendon xanthomas. None of the family members mentioned earlier had arcus cornea. The paternal grandmother of the proband died at the age of 41 years from myocardial infarction, whereas the mother, brother, and grandfather of the proband had no hypercholesterolemia. The family tree of the proband is shown in Figure 1B, and the results of lipid analyses before treatment are shown in Table 1. Based on the proband’s family history, physical examination, lipids results, and subsequent DNA analyses, the proband was diagnosed with FH. According to the statement of the proband, statins were occasionally used before the diagnosis. After the diagnosis, atorvastatin (20 mg per night) and ezetimibe (10 mg per night) were prescribed, and the proband was followed in the clinic each month for 6 months. The serum TC and LDL-C varied between 6–7 and 4–5 mmol/L during follow-up.

### Identification of Genomic Variants

**FIGURE 2:**
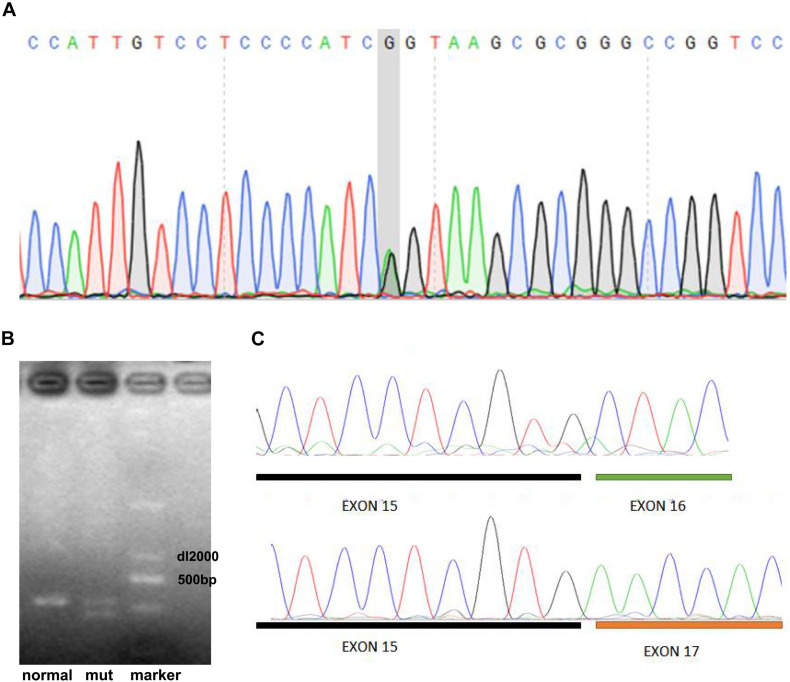
Identification of genomic variant in *LDLR* of proband. **(A)** DNA sequencing showed a single-base substitution (G < A) at position 2389 in 16th exon of *LDLR* gene. **(B)** RT-PCR including 16th exon showed that proband had an additional PCR product of a different length from one PCR product obtained for healthy control. **(C)** Sequencing results showed that shorter PCR product exclusively observed in proband had deletion of 16th exon of mutant *LDLR*.

Given that functional genomic variants in *LDLR*, *PCSK9*, and *APOB* are the main causes of FH (Henderson et al., 2016; Sharifi et al., 2017), we performed target exome capture-based next-generation sequencing of these genes to narrow down the potential genomic variants in this case of FH. Our genomic DNA sequencing found no genomic variants in the *PCSK9* and *APOB* genes. However, there was a single-base substitution (G < A) at position 2389 in the 16th exon of the *LDLR* gene as shown in Figure 2A, which would lead to a change from valine (V) to methionine (M) at the protein sequence of 797 (V797M), if the protein is translated successfully. However, the genomic variants (c. 2389G > A) was located in the last base of exon 16, which led to the hypothesis that the variant in this case of FH may have a potential influence on mRNA cleavage progression. To test this hypothesis, we further performed quantitative reverse transcription PCR of peripheral venous blood samples from the proband and a healthy control to identify differences in the *LDLR* mRNA. With the primers for the 16th exon, the results showed that the proband had an additional PCR product of a different length from the one PCR product obtained for a healthy control subject (535 bp, Figure 2B). The sequencing results showed that the shorter PCR product exclusively observed in the proband had a deletion of the 16th exon of the mutant *LDLR* (Figure 2C), indicating that c. 2389G > A in *LDLR* disrupted normal mRNA transcription *via* disturbing mRNA cleavage. Genomic DNA sequencing by Sanger sequencing showed a similar variant (c. 2389G > A) in the family members with FH (father, paternal uncle, and aunt, Table 1) but not in the family members without FH (mother and brother).

### Cellular Localization of Mutant LDLR Protein

**FIGURE 3:**
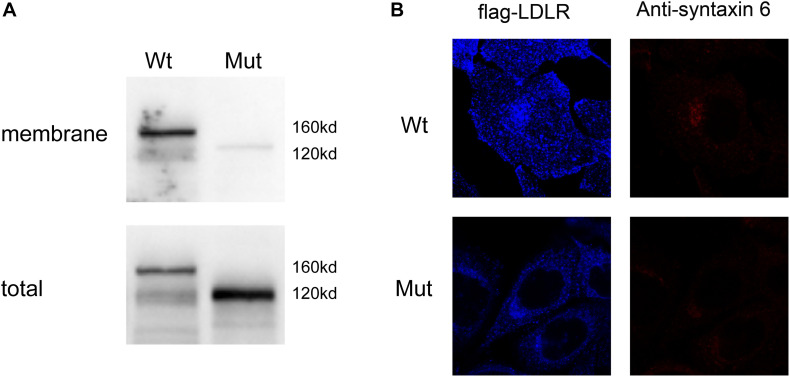
Expression of different LDLR variants on cell surface of HepG2-transfected cells. **(A)** Western blot analysis showed that mutant LDLR protein with exon 16 deletion was rarely expressed on membrane but accumulated in plasma of HepG2 cells with a molecular weight of 120 kDa. Membranous LDLR was analyzed by biotinylation as described in section “Analysis of Surface Biotinylated Proteins and Western Blotting.” **(B)** CLSM examination showed that WT LDLR was mainly distributed at cell surface, whereas mutant LDLR with exon 16 deletions was mainly retained in Golgi apparatus. Blue fluorescence represents distribution of Flag antibody. Because Flag gene and LDLR gene are fused, blue fluorescence distribution reflects distribution of LDLR protein in cell (LDL-R). Red fluorescence indicates the position of Golgi apparatus (marked with anti-syntaxin 6).

To further characterize the localization of the mutant LDLR protein, we constructed viral vectors of *LDLR* with exon 16 deletion, and WT *LDLR* with FLAG merged on the C-terminus and infected the conventional cell line of human liver cells. We then used Western blot analyses to evaluate the amount of LDLR protein on the membrane and among total cellular proteins. As shown in Figure 3A, opposite to the considerable membrane expression of LDLR as detected by Western blotting on HepG2 cells transfected with WT *LDLR*, LDLR protein was barely detected on the membrane cells transfected with the mutant *LDLR* with exon 16 deletion. However, a considerable amount of LDLR at 120 kDa was detected when the total protein was used for Western blot analysis in the mutant cells, indicating the accumulation of an LDLR precursor-like protein in the plasma of the HepG2 cells with mutant LDLR. To further determine the cellular localization of the LDLR-FLAG fusion proteins, a CLSM examination was used. To our surprise, WT LDLR was mainly distributed at the cell surface. However, the mutant LDLR with exon 16 deletion was retained primarily in the Golgi apparatus (Figure 3B). Taken together, these results indicate that LDLR exon 16 deletion led to LDLR protein re-localization in the Golgi apparatus rather than at the cell surface.

### Reduced 1,19-Dioctadecyl-3,3,3939-Tetramethylindocarbocyanine Perchlorate-Low-Density Lipoprotein Uptake Ability in HepG2 Cells Transfected With Mutant *LDLR* Gene

**FIGURE 4:**
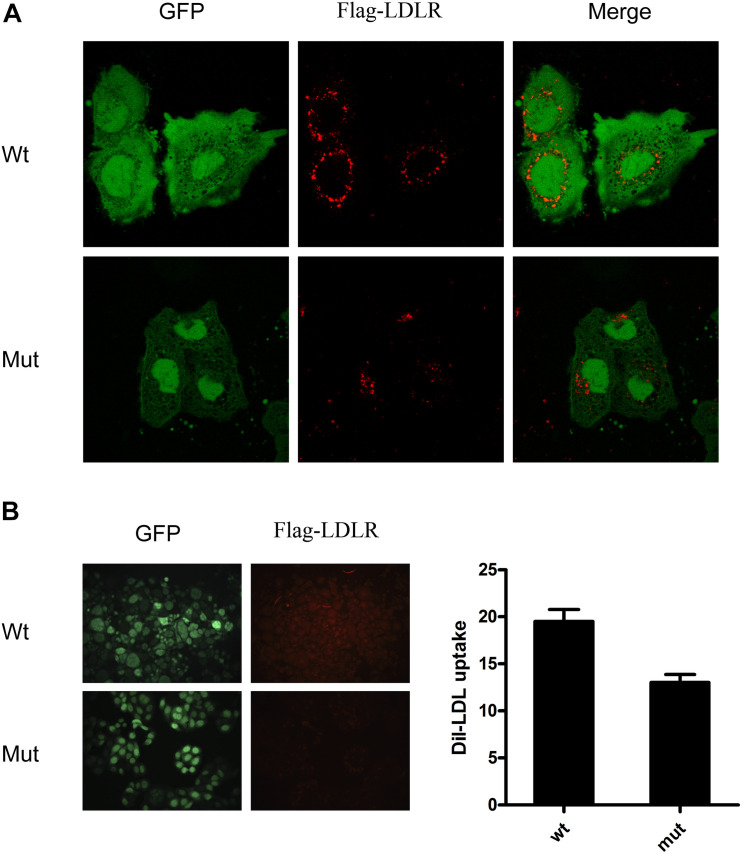
DiI-LDL uptake measurement showed that internalization capacity of HepG2 cells with mutant LDLR with exon 16 deletion was substantially lower than that observed in cells with WT LDLR. **(A)** Red fluorescence represents DiI-LDL, and green fluorescence represents HepG2-transfected cells. **(B)** Quantitative analysis showed that capacity of DiI-LDL uptake was significantly reduced in HepG2 cells transfected with c. G < A 2389 that resides in 16th exon of LDLR as compared with HepG2cells transfected with WT LDLR.

To evaluate the functional consequence of mutant LDLR with exon 16 deletion, we used DiI-LDL to evaluate the uptake ability of the LDLR in HepG2 cells. We observed that the internalization capacity of HepG2 cells with mutant LDLR was substantially lower than that of cells with WT LDLR (13.6 vs. 19.5%, *P* < 0.05; Figure 4). From their results taken together, this variant was classified as likely pathogenic with autosomal dominant inheritance by the American College of Medical Genetics and Genomics. Because this variant has been reported in individuals with FH in various studies^1^, this variant has been found in ≥ 10 unrelated FH cases (PS4). Moreover, our functional analysis fulfilled PS3_Moderate (1) and (3) criteria.

## Discussion

In this study, we identified and characterized a single-base substitution genomic variant (c. G < A 2389) in a Chinese proband with FH. Subsequent functional characterization of the variants showed that c. G < A 2389 resides in the 16th exon of LDLR and leads to the miscleavage of LDLR mRNA and loss of the 16th exon. Accordingly, exon 16 deletion occurred in the mutant LDLR protein, which resulted in the retention of the mutant LDLR in the Golgi apparatus, reduced expression of LDLR on the cellular membrane, and substantially reduced uptake ability of the LDLR protein for LDL-C.

Physically, LDLR is known as a type 1 transmembrane protein that functions by binding and internalizing LDL-C and mediating its subsequent release and degradation in the endosomes and lysosomes (Willnow, 1999; Defesche, 2004). LDLR is initially synthesized as a precursor of 860 amino acids (AAs), including a signaling peptide of 21 AAs. After cleavage of the signaling peptide, the mature LDLR protein with 839 AAs is transported into the ER, where it undergoes folding and glycosylation. Post-translational modification of LDLR also occurs in the Golgi apparatus, likely because LDLR requires O-glycosylation for its stable expression and incorporation into the cellular membrane, where clathrin-coated pits form to confer the internalization and subsequent intracellular metabolism of LDL-C. Five functional domains have been identified in the LDLR protein, and the transmembrane domain that consists of AAs 789–810 is considered to be evolutionarily preserved and to play a fundamental role in the incorporation of the protein into the cellular membrane (Willnow, 1999; Defesche, 2004; Mahdieh et al., 2020). The results of our sequencing analyses for the *LDLR* gene and its mRNA indicated the loss of exon 16 caused by the single-base substation genomic variant (G < A 2389), which led to coding of the last base of the 15th exon (2311) and the first two bases of the 17th exon (2390 and 2391). Accordingly, mutant LDLR with exon 16 deletion was formed, and the original 789–769 AAs of the transmembrane domain of the LDLR protein were missing, which finally led to the failure of the mutant LDLR to incorporate into the cellular membrane. From a search of the clinical variation database, this variant has been reported in individuals with FH in various studies (see text footnote 1). This variant has been found in several FH patients, along with evidence of co-segregation with the disease. Multiple clinical diagnostic laboratories/reputable databases have classified this variant as likely pathogenic/pathogenic. The variant was first reported by Mak et al. (1998) in a Chinese patient with FH. They found that G < A 2389 occurs at the last base of exon 16, which may be the −1 position of the 5′ donor splice site. Reverse transcription PCR investigated its effect on splicing. Only the normal G allele was found. This mutation thus appears to cause a donor site splicing error for the LDL receptor gene (Mak et al., 1998). However, functional analysis has not been reported, making the present analysis valuable in understanding this genomic variant. Our functional studies, as complementation, showed that c. G < A 2389 resides in the 16th exon of LDLR and leads to the miscleavage of LDLR mRNA and loss of the 16th exon. Accordingly, exon 16 deletion occurred in the mutant LDLR protein, which resulted in the retention of the mutant LDLR in the Golgi apparatus, reduced expression of LDLR on the cellular membrane, and substantially reduced uptake ability of the LDLR protein for LDL-C.

The mechanisms underlying the retention of the mutant LDLR in the Golgi apparatus are not fully understood. Considering the importance of O-glycosylation of the LDLR protein for its stable expression and incorporation into the cellular membrane and the fact the molecular weight of the mutant LDLR protein is similar to that of the precursor of LDLR (120 kDa), we hypothesized that the glycosylation process might also be impaired due to the genomic variants, causing retention of the mutant LDLR in the Golgi apparatus. Interestingly, no previous study has reported any genomic variants of *LDLR* that cause retention of the mutant LDLR protein in the Golgi apparatus. In fact, a recent study reported a p.L799R variant in *LDLR* that leads to the translocation of the entire L799R-LDLR into the lumen of the ER (Strom et al., 2015).

In conclusion, we identified a single-base substitution variant (g. G < A 2389) in a Chinese proband with FH, which is associated with miscleavage of LDLR mRNA and subsequent retention of the protein in the Golgi apparatus. Further studies are needed to determine the exact mechanisms underlying the pathogenic effects of this genomic variant in FH, which may be important for better understanding the pathogenesis of FH.

## Data Availability Statement

The raw data supporting the conclusions of this article will be made available by the authors, without undue reservation.

## Ethics Statement

The studies involving human participants were reviewed and approved by Ethic Committee of The Affiliated Hospital of Qingdao University. The patients/participants provided their written informed consent to participate in this study. Written informed consent was obtained from the individual(s) for the publication of any potentially identifiable images or data included in this article.

## Author Contributions

H-YS and Y-GW designed the study. Y-CL and C-CZ were involved with patient care. H-YS analyzed patient samples. WZ advised on histological staining and analysis. Y-CL and C-CZ assisted with sample analysis. H-YS and WZ drafted and wrote the manuscript. Y-GW, Y-CL, and C-CZ revised the manuscript critically for intellectual content. Y-GW had primary responsibility for the final content. All authors read and approved the final manuscript.

## Conflict of Interest

The authors declare that the research was conducted in the absence of any commercial or financial relationships that could be construed as a potential conflict of interest.

## Publisher’s Note

All claims expressed in this article are solely those of the authors and do not necessarily represent those of their affiliated organizations, or those of the publisher, the editors and the reviewers. Any product that may be evaluated in this article, or claim that may be made by its manufacturer, is not guaranteed or endorsed by the publisher.

## Footnotes

1
https://www.ncbi.nlm.nih.gov/clinvar/variation/226393/

## Supplementary Material

Supplementary Figure 1 Full gel image from RT-PCR including the 16th exon showing that the proband had an additional PCR product of a different length from the single PCR product obtained for the healthy control.

Supplementary Figure 2 Full gel image of western blot analysis showing that the mutant LDLR protein with exon 16 deletion was rarely expressed on the membrane, but accumulated in the plasma of the HepG2 cells with a molecular weight of 120 kDa.
