# Supplementary material for: Identification and Functional Characterization of a Low-Density Lipoprotein Receptor Gene Pathogenic Variant in Familial Hypercholesterolemia
Source: Front Genet. 2021 Aug 23;12:650077. doi: 10.3389/fgene.2021.650077 (PMC8419346; doi:10.3389/fgene.2021.650077)

Supplementary Material

## Supplementary Figures

**Supplemental Figure 1.** Full gel image from RT-PCR including the 16th exon showing that the proband had an additional PCR product of a different length from the single PCR product obtained for the healthy control.

**
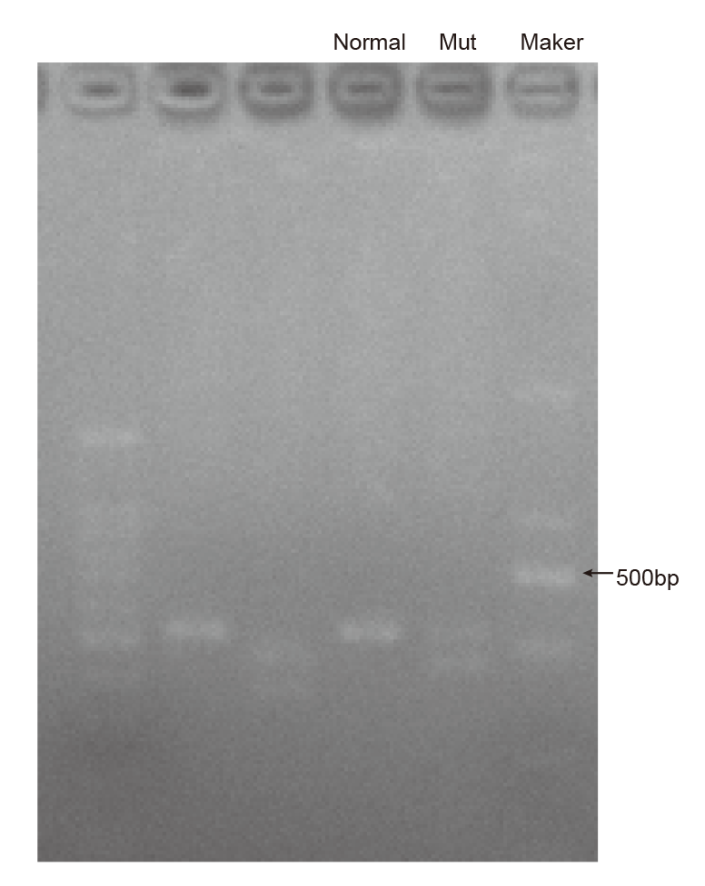
**

**Supplemental Figure 2.** Full gel image of western blot analysis showing that the mutant LDLR protein with exon 16 deletion was rarely expressed on the membrane, but accumulated in the plasma of the HepG2 cells with a molecular weight of 120 kDa.


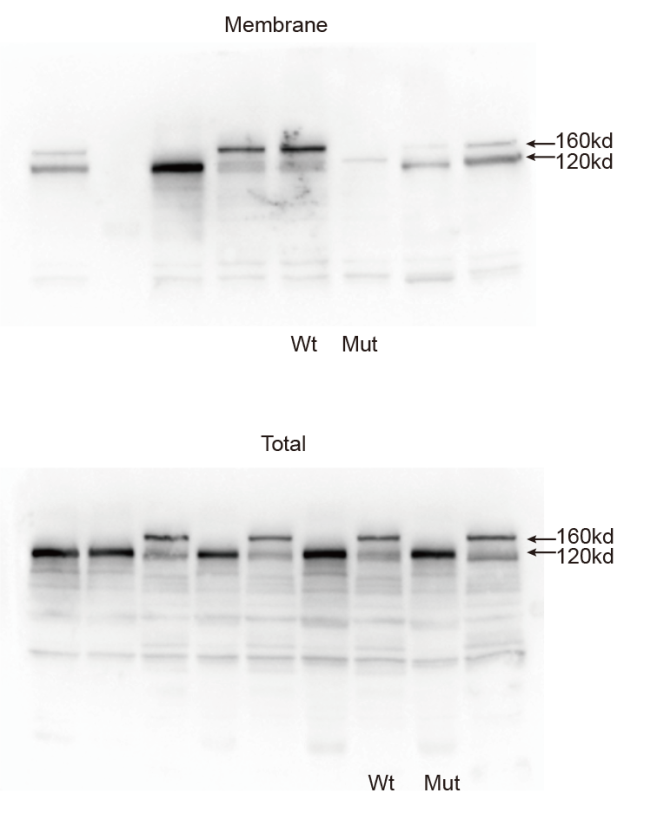

Supplement: Supplementary Figure 1 — Full gel image from RT-PCR including the 16th exon showing that the proband had an additional PCR product of a different length from the single PCR product obtained for the healthy control. [file Data_Sheet_1.docx]
